# Mathematical Modeling and Simulation Provides Evidence for New Strategies of Ovarian Stimulation

**DOI:** 10.3389/fendo.2021.613048

**Published:** 2021-03-11

**Authors:** Sophie Fischer, Rainald Ehrig, Stefan Schäfer, Enrico Tronci, Toni Mancini, Marcel Egli, Fabian Ille, Tillmann H. C. Krüger, Brigitte Leeners, Susanna Röblitz

**Affiliations:** ^1^ Computational Biology Unit, Department of Informatics, University of Bergen, Bergen, Norway; ^2^ Computational Systems Biology Group, Zuse Institute Berlin (ZIB), Berlin, Germany; ^3^ Department of Microstructure and Residual Stress Analysis, Helmholtz Centre Berlin for Materials and Energy, Berlin, Germany; ^4^ Department of Computer Science, University of Rome “La Sapienza”, Rome, Italy; ^5^ Centre of Competence in Aerospace Biomedical Science & Technology, Lucerne University of Applied Sciences and Arts, Lucerne, Switzerland; ^6^ Department of Psychiatry, Social Psychiatry and Psychotherapy, Hannover Medical School, Hannover, Germany; ^7^ Department of Reproductive Medicine, University Hospital Zurich, Zurich, Switzerland

**Keywords:** endocrinological networks, systems biology, follicular dynamics, ordinary differential equations, assisted reproductive technologies

## Abstract

New approaches to ovarian stimulation protocols, such as luteal start, random start or double stimulation, allow for flexibility in ovarian stimulation at different phases of the menstrual cycle. It has been proposed that the success of these methods is based on the continuous growth of multiple cohorts (“waves”) of follicles throughout the menstrual cycle which leads to the availability of ovarian follicles for ovarian controlled stimulation at several time points. Though several preliminary studies have been published, their scientific evidence has not been considered as being strong enough to integrate these results into routine clinical practice. This work aims at adding further scientific evidence about the efficiency of variable-start protocols and underpinning the theory of follicular waves by using mathematical modeling and numerical simulations. For this purpose, we have modified and coupled two previously published models, one describing the time course of hormones and one describing competitive follicular growth in a normal menstrual cycle. The coupled model is used to test ovarian stimulation protocols *in silico*. Simulation results show the occurrence of follicles in a wave-like manner during a normal menstrual cycle and qualitatively predict the outcome of ovarian stimulation initiated at different time points of the menstrual cycle.

## Introduction

Infertility is a worldwide problem. According to the World Health Organization, about 48.5 million couples worldwide were affected by unwanted childlessness in 2010, and the number continues to grow (1). Men and women are just as likely to contribute to the couple’s infertility (2). Infertility as a disease of the female reproductive system affects approximately 10% of women of reproductive age worldwide (3). Unbalanced hormone levels are one cause, in a wide range of conditions, leading to infertility. For many couples, unwanted childlessness is a burden. Assisted reproductive technologies (ART) provide strategies to deal with infertility. Both unwanted childlessness and ART increase the risk for negative psycho-social functioning, such as depression and anxiety disorders (4–6), whereby the treatment burden has fallen mainly on women (2). Therefore, new ART approaches deserve to be highlighted. We want to add further scientific evidence for the efficiency of those new approaches by using mathematical modeling and numerical simulations.

**Figure 1:**
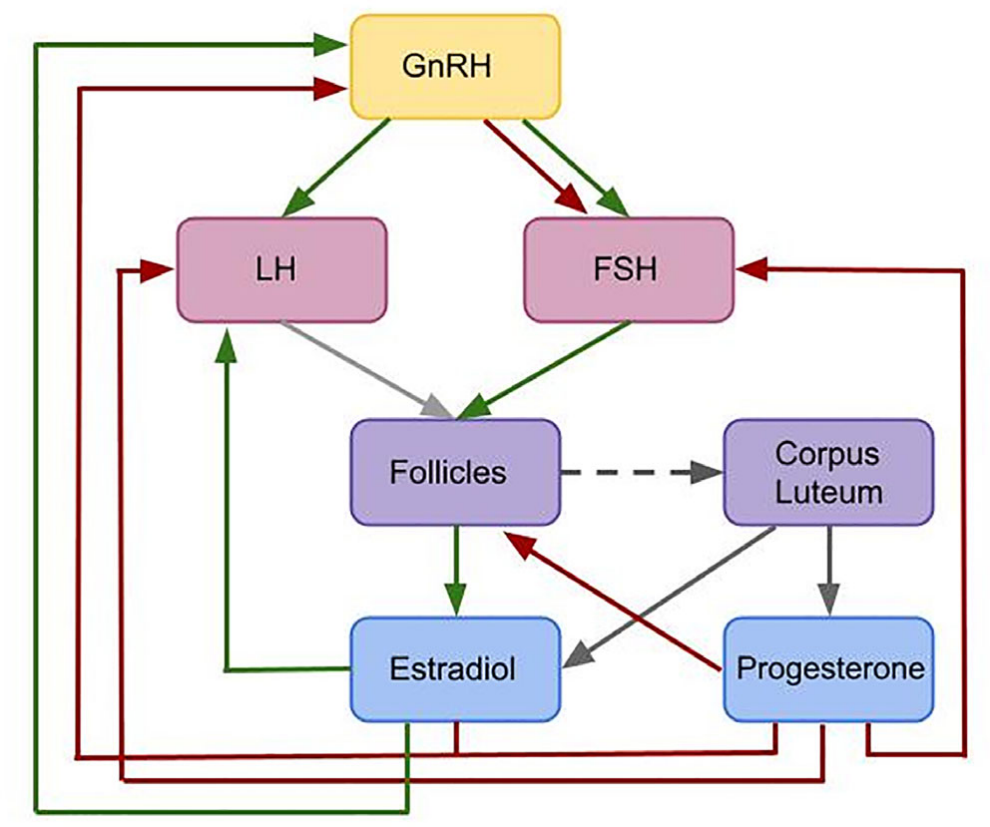
Flowchart illustrating the interactions included in the given model. This is a simplified feedback interactions network for the hormonal control of the female menstrual cycle. Green arrows indicate positive feedback effects, while red arrows express negative feedback interactions. Gray arrows show other types of interactions. The pulsatile release of GnRH stimulates the release of the pituitary hormones LH and FSH. These hormones effect follicular maturation. Growing follicles produce E2 which has a positive feedback effect on the LH concentration. A high LH concentration triggers the ovulation of one selected follicle (light gray arrow) followed by the formation of the corpus luteum (dark gray dashed arrow). The simultaneous release of E2 and P4 by the corpus luteum (dark gray arrows) inhibits the release of GnRH. Additionally, P4 has an inhibitory effect on LH and FSH. While P4 only has an inhibitory effect on GnRH, E2 has either a stimulatory or an inhibitory effect on GnRH, depending on the E2 level.

Female reproduction is essentially enabled by a feedback mechanism between ovarian hormones, mainly progesterone (P4) and estradiol (E2), and the pituitary hormones luteinizing hormone (LH) and follicular stimulating hormone (FSH), see **Figure 1**. The hormone interaction network is important for regulating folliculogenesis. While the initial recruitment of follicles does not depend on gonadotropins (7, 8), the growth of cohorts of larger follicles relies on a stimulatory effect of FSH. FSH signaling is mediated by the expression of FSH receptors on granulosa cells (9, 10). The gonadodropins LH and FSH are responsible for follicular estradiol production. LH stimulates androstenedione production, which is the substrate for the FSH stimulated aromatase reaction producing estradiol (8, 11, 12). Around mid-cycle, usually one dominant follicle ovulates and releases an oocyte. The remaining parts of the dominant follicle transform into the corpus luteum, which has a key role in preparing the body for a possible pregnancy. If the oocyte is not fertilized, the corpus luteum decays and a new cycle starts (13–15). Interruptions in the feedback system are one reason for infertility.

Modern assisted reproductive technologies like *in vitro* fertilization (IVF) or intracytoplasmic sperm injection (ICSI) have increased the chance for pregnancy. Ovarian stimulation, which aims at obtaining multiple fertilizable oocytes, is a critical step in ART (16). Since the 1980s, the long gonadotropin-releasing hormone (GnRH) agonist protocol has been commonly used to prepare for oocyte retrieval and in-vitro fertilization (17, 18). This protocol starts around mid-luteal phase with GnRH agonist administration for about 14 days. Right after the beginning of GnRH agonist administration, a short period of gonadotropin (FSH and LH) hypersecretion is observable. The treatment leads to GnRH-receptor down-regulation in the pituitary (19, 20). In the next step, the growth of multiple follicles is stimulated by FSH administration alone, e.g. with recombinant FSH (rFSH), or by a combination of FSH and LH, e.g. with human menopausal gonadotropin (hMG). Continuation of GnRH agonist administration during the stimulation phase prevents an LH surge and hence ovulation. In the final step, ovulation is induced by injecting human chorionic gonadotropin (hCG) (18). Patient-specific and clinic-dependent modifications of these general procedures are common. The two most common alternatives are the short GnRH agonist protocol and the antagonist protocol. Both protocols work without downregulation, though some clinics perform a pre-treatment phase for 10 to 25 days with a P4 antagonist that inhibits ovulation.

The stimulation phase in the short GnRH agonist protocol is the same as in the long protocol. It includes the stimulation with hMG or rFSH and the concurrent administration of a GnRH agonist. The antagonist protocol also includes the stimulation with hMG or rFSH but, in contrast to the agonist protocols, a GnRH antagonist is administered from day 5 of the stimulation period. The final step in all protocols is the induction of ovulation by hCG.

In general, infertility treatment is a long-term and expensive therapy with high dropout rates (21), mainly because it imposes physical, mental, and emotional burdens (22). Often, life has to be subordinated to medical procedures. Therefore, treatment alternatives are of interest. Both the short and the antagonist protocol are less time-consuming than the long protocol. However, the stimulation phase in these protocols conventionally starts in the early follicular phase. This constraint could cause too long waiting times, e.g. for women requiring emergency fertility preservation. Hence, the advancement of a new class of ovarian stimulation approaches called random - and luteal phase-start ovarian stimulation protocol (23) has progressed. In recent years, several studies investigating ovarian stimulation protocols with various starting points have been published (24–26). Originally, these protocols were invented for fertility preservation in cancer patients, where time is a limiting factor (27). However, they might be beneficial for patients outside an oncological setting (23), though there is an ongoing debate whether the oocyte quality differs between protocols. Other approaches like the double ovarian stimulation, where two waves within one cycle are stimulated, might help to increase the number of accumulated oocytes within one treatment cycle (28). That strategy could be of particular interest for the therapy of poor ovarian response patients (29, 30).

One possible explanation for the success of stimulation initiated in different phases of the cycle is the “wave” theory. The use of high-resolution transvaginal ultrasonography has underpinned the hypothesis that, similar to ruminants, follicular growth and development in human is characterized by waves (31, 32), whereby each wave involves the recruitment of a cohort of follicles and the possible selection of a dominant follicle. Given that multiple waves of follicles appear each cycle, there are multiple time points during one cycle that are suitable to start ovarian stimulation.

The mathematical model underlying this study simulates the time-evolution of key hormones and growth behavior of multiple follicles. In particular, we test the hypothesis that random recruitment of follicles leads to the emergence of follicular waves. Based on the occurrence of follicular waves that we observe in our simulation results, we study variable-start ovarian stimulation protocols in silico. We demonstrate simulation results for two protocols, namely (i) stimulation initiated in the late follicular phase and (ii) stimulation initiated in the luteal phase. We analyze statistics of treatment duration and numbers of follicles in our simulation results and compare them with the literature.

## Materials And Methods

### Mathematical Modeling of the Female Menstrual Cycle

Mathematical modeling is a useful tool to better understand the human menstrual cycle by validating or testing hypothesis in silico, and predicting possible dynamics. A first mathematical model for the human menstrual cycle was introduced in a series of articles by Schlosser, Selgrade, and Harris-Clark (33). Their model allows to simulate the time course of hormones and follicular maturation stages over several cycles and is able to display multiple follicular waves (34). This model was extended by pharmacokinetic sub-models to simulate the administration of drugs, including ovarian contraceptive pills (35, 36) and GnRH analogs (37). These pharmacokinetic-pharmacodynamic (PKPD) models allow to study the influence of dose and time point of administration of various drugs on the cycle dynamics.

All those models are based on ordinary or delay differential equations since they allow to simulate the time evolution of hormone concentrations and follicles. Hill functions have been used to characterize stimulatory and inhibitory effects, as it is common practice for modeling regulatory networks. The model by Röblitz et al. (37) consists of 33 ordinary differential equations that describe the feedback mechanisms between the hormones that are of particular importance for the female menstrual cycle (GnRH, FSH, LH, E2, P4, inhibin A, inhibin B) and the development of follicles and corpus luteum throughout consecutive cycles. Compared to previous models, it does not use delay differential equations and consists of fewer equations and parameters. However, all those models have in common that follicular growth is described in terms of activity levels of different follicular maturation stages, but not in terms of follicle numbers and sizes. Thus, the simulation results cannot be compared with ultrasound data.

A mathematical model that quantifies the time evolution of the sizes of multiple follicles comparable to observations by ultrasound measurements in mono-ovulatory species was presented by (38). This model contains a separate differential equation for each follicle, whereby the structure of this equation is the same for all follicles, but the initial follicle sizes are different. The equations are coupled *via* a term that accounts for competitive interactions between follicles. Together with the model by (37) a previous version of the model by (38) formed the basis for the development of computational tools to enable in silico clinical trials in reproductive endocrinology (39, 40). In particular, by introducing variability into model parameters (41–43), the authors could analyze inter-individual variability in the cycle and automatically synthesize, by means of artificial intelligence guided by patient digital twins, optimal personalized treatments for the patients at hand (44). However, the tools could only be applied to the downregulation phase before follicular stimulation, because the feedback mechanisms from the ovaries to the pituitary were not implemented in the modified model. This drawback motivated the development of the fully coupled model as presented in this work. To our knowledge, this is the first mathematical model that allows for the simulation of stimulation protocols that start at different time points in the cycle.

#### Model Construction and Assumptions

The mathematical model underlying this work is the result of modifying and coupling the two previously published models by Röblitz et al. (37) and Lange et al. (38). In a first step, the model by Röblitz et al. (37) was reduced by removing the equations for the development of follicles and the corpus luteum and the hormones produced by them (inhibin A, inhibin B, E2, P4). In addition, we removed the equations for LH receptor binding mechanisms, since they were not needed for our purpose. The remaining equations were kept exactly as in (37), except for the FSH synthesis rate. In the new model, this rate is inhibited by P4 instead of inhibin A and B [**Eq. (S5) in the Supplement**], since P4 reaches its peak in the mid-luteal phase exactly as inhibin A. The influence of inhibin B could be neglected without any consequences for the qualitative behavior of the model. In addition, we have introduced a new equation for the amount of FSH that reaches the follicles [**Eq. (S9) in the Supplement**] to account for delays caused by transportation and for changes in concentration caused by different volumes. In contrast to (37), the equations for FSH receptor binding now take into account FSH in the ovaries instead of the FSH blood concentration [**Eqs. (S10)–(S12) in the Supplement**].

Instead of re-introducing a corpus luteum into the model equations, we decided to use algebraic equations to directly model the amounts of E2 and P4 produced in the luteal phase of the cycle [**Eqs. (S23) and (S25) in Supplement S1**]. The model describes E2 and P4 levels in the luteal phase by Gaussian-shaped curves with fixed parameters based on fits to experimental data (for P4 see **Figure S1 in Supplement S3**). This simplification is based on the observation that the variability in the length of the luteal phase is significantly lower than the variability in the length of the follicular phase (45).

We modified the follicle equation introduced by (38) in a way that the hormone dynamics in the system have a direct effect on the follicular growth behavior [**Eqs. (S20)-(S22) in Supplement S1**]. The maturation of each follicle is modeled by a separate ODE. All ODEs have the same structure and include both shared and follicle specific parameters. Each follicle carries two random properties that are follicle specific, hence there are two follicle specific parameters: the time point at which a follicle is recruited, and its FSH sensitivity. The following assumptions are made about these two parameters:

- The time point at which a follicle is recruited and starts growing is follicle-specific and follows a Poisson process. The overall number of follicles that are recruited within a specific time interval is a Poisson random variable. The parameter of this distribution, in the following named Poisson parameter, corresponds to the probability that a given number of follicles is recruited in a fixed time interval. In the model, the Poisson parameter is modulated by the FSH concentration: if the FSH concentration is above a certain threshold, more follicles are recruited.
- The second property is a follicle specific FSH value, referred to as FSH sensitivity threshold value, which has to be exceeded in order to stimulate the follicle’s growth. This refers to the biological finding that follicle growth does not occur below a certain level of FSH (46), and that any two follicles might respond differently to FSH, even if the two have the same size, because they differ in the FSH receptor density. The distribution of the FSH sensitivity threshold values in the population of follicles is assumed to follow a normal distribution. Follicles that are more sensitive to FSH, i.e. which require less FSH to start growing, have a competitive advantage for being selected as the dominant follicle. Whether a follicle becomes dominant, however, depends on both its FSH sensitivity and its recruitment time point.

The competition between follicles, which is represented by a common parameter [**Eq. (S22) in Supplement S1**], is inhibited by FSH concentrations above a certain threshold, taking into account the “FSH window concept” (47–49). This concept is based on the observation that the period of time during which FSH is above a certain threshold effects the number of follicles reaching the dominant follicle’s size (50, 51). Moreover, we assume that the follicular growth rate is inhibited by P4 and stimulated by the FSH receptor complex level in a threshold dependent way [**Eq. (S21) in Supplement S1**] (52).

Growing follicles are the main source of E2 in the female body and the dominant follicle produces the most E2 (12, 53, 54). Estradiol is produced by granulosa cells, which proliferate and form a multilayered structure. This is included in the model by an additional term in E2 production which is dependent on the follicular size [**Eqs. (S24) and (S25) in Supplement S1**].

To sum up, the coupling between the hormone dynamics model and the follicular growth model is realized as follows (compare **Figure 1**). The levels of FSH in the blood and of the FSH receptor complex enter into the equations for the follicles in a threshold dependent way. In addition, the LH level plays a role in determining the time point of ovulation. Ovulation of a follicle that exceeds the size threshold occurs 12 h after the LH level is higher than a certain threshold. The levels of E2 and P4 in the luteal phase depend on the time point of the last ovulation. E2 and P4 levels enter into the equations for LH and FSH synthesis and for the frequency and mass of GnRH, in the same way as in (37). The coupled model contains in total 72 parameters, i.e. less than the two original models taken together (114 parameters in (37) and 5 parameters in (38). We adopted 44 parameters from (37) and only changed the values of three of them. A detailed parameter list can be found in the Supplement. The model has been implemented in MATLAB and numerical simulations were performed using the ODE solver *ode15s*. The code is available at https://github.com/SoFiwork/GynCycle.

### Ovarian Stimulation Protocols

Stimulation protocols are introduced to the model by a pharmacokinetic approach. The dosing concentrations of the administered drug, as used in the ovarian stimulation protocols, are calculated during the simulation based on three drug specific pharmacokinetic parameters using the information given by (55) [**Eq. (S26) in Supplement S1**]. In order to study treatment outcomes, two different stimulation protocols were implemented. The two studies were selected based on the accessibility of results, the size of study cohorts and the physiological stage of patients. Each study includes data from more than 100 women. Patients were at the age of 18 to 40 years with a body mass index of 18 to 30 kg/m^3^. All women showed spontaneous ovulation.

#### Stimulation Initiated in the Late Follicular Phase

Our simulated treatment protocol for ovarian stimulation during the late follicular phase follows the description in Zhu and Fu (24). As a simplification, we did not vary the administered hMG dose during the first days of stimulation. The stimulation starts with a daily administration of 150 IU hMG when at least one follicle measures 14 mm in diameter. After 6 days, the daily dose is increased to 225 IU per day. We chose day 6 to change the hMG concentration because re-examination and dose adjustment in the clinical trial took place after 5 - 7 days. The stimulation stops whenever at least 3 follicles reach a diameter of at least 18 mm. The ovulation of a dominant follicle during the stimulation phase is characteristic for this protocol.

#### Stimulation Initiated in the Luteal Phase

The protocol described in (26) served as a reference to simulate the stimulation of multiple follicular growth during the luteal phase. In this clinical trial, the drug administration in the simulation starts between day 1 and 3 after ovulation under the condition that there exist follicles smaller than 8 mm. Follicular growth is stimulated by the daily administration of 225 IU hMG. The stimulation terminates if at least three follicles have reached a diameter of 18 mm.

## Results

### Unstimulated Cycle

**Figure 2:**
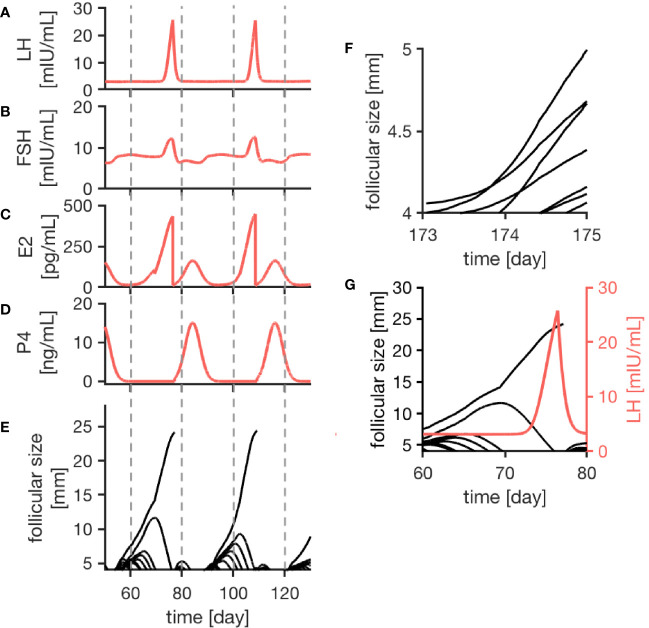
Simulation results of the female menstrual cycle model are displayed. The left column illustrates the simulation outcome for two menstrual cycles and the right column zooms into details. Here, one cycle is defined from one ovulation to the next one. Sub-figures **(A–D)** represent the simulated hormone concentration profiles for LH, FSH, E2 and P4. **(E)** portrays growth trajectories of follicles >4 mm. The ovulation of a dominant follicle is indicated by terminating trajectories, as seen for example around day 80 of the simulation. **(F)** illustrates competition between follicles indicated by crossing growth trajectories. **(G)** Points out that the ovulation of a dominant follicle occurs 12 h after the LH peak concentration as a result of the way the ovulation process is implemented in the model.

As indicated in **Figure 2**, the model generates quasi-periodic solutions for all four hormones. Due to the individual growth behavior of follicles implemented in the model, variations in cycle length and number of follicles per cycle occur. Simulations for a normal cycle were performed for more than 1000 time steps in order to get an idea of the variability in the model outcome. In total, 42 simulated menstrual cycles (here, one menstrual cycle is defined from one ovulation to the next one) were used for a statistical analysis. In the simulations, the average cycle length was 30.56 days, with a standard deviation of 7.00 days (**Figure S2 in Supplement**). On average, 16.19 follicles greater than 4 mm were detected during one cycle, with a standard deviation of 3.08 follicles. The results were tested for normality using the Shapiro-Wilk test with a 95 confidence interval. A correlation between the cycle length and the follicular count was not observed.

The simulated hormone curves are supposed to be comparable to serum hormone concentration profiles in terms of shape and peak values. **Figures 2A–E** display consecutive menstrual cycles in the time period between day 50 and day 130 from one simulation run. The time evolution of all four hormone profiles is illustrated, and the described interplay between hormones and follicles is apparent.

The wave-like growth behavior of the follicles (**Figure 2E**) is generated by the model itself and is not enforced by the implementation. **Figure 2G** shows an example of the ovulation of a dominant follicle that occurs 12 h after LH reached its peak concentration. This 12-h gap is accomplished by the way the ovulation event is defined in our model (see *Discussion*). Once ovulation is detected during the run time of the simulation, the ovulated follicle is taken out from the cohort of follicles (indicated by the terminating trajectory in **Figure 2G**). This follicle no longer contributes to steroid production. Keeping it in the simulation would needlessly increase computational time. The growth behavior of follicles causes variation in the length of the follicular phase. In contrast to that, the luteal phase has a constant length of 14 days due to its implementation.

The follicular growth equation, as introduced by (38) and modified for the given model, includes a term addressing the competition for dominance between follicles. In the simulation results, its effect is visible by crossing growth trajectories (**Figure 2F**). This crossing only is possible because each follicle has its specific parameters. As it can be seen in **Figure 2**, competition is stronger during the early follicular phase before a dominant follicle emerges.

### Ovarian Stimulation

The simulations of ovarian stimulation initiated in the luteal phase or the late follicular phase are characterized by the growth of multiple follicles. Additionally, the ovulation of a dominant follicle during a stimulation protocol occurs only during stimulation in the late follicular phase. In the model, the competition term is inhibited by high FSH concentrations, enabling the growth of multiple follicles under stimulatory treatment.

**Figure 3:**
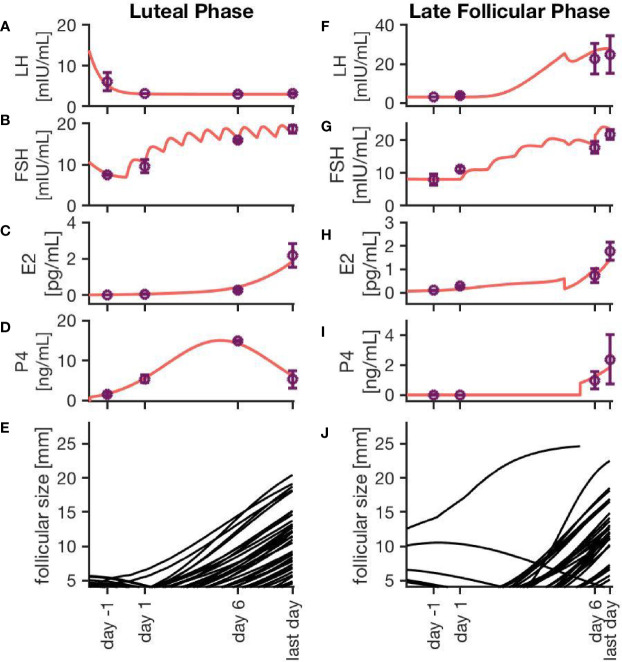
Simulation results for two different ovarian stimulation protocols. The growth of multiple large follicles, caused by the stimulation treatment, is characteristic for both strategies. The left column represents simulation results from a luteal phase stimulation protocol, while the right column shows the effect of a stimulation during the late follicular phase. Sub-figures **(A–D, F–I)** exemplary represent hormone profiles originating from one simulation in red. Purple dots and error bars represent mean values and variances, respectively, from 20 simulations at four characteristic time points: 1 day before the stimulation treatment starts, 1 day after starting the treatment, 6 days after starting the treatment, and the last day of treatment. Sub-figures **(E, J)** illustrate the growth trajectories of the follicles.

**Figure 3** exemplarily displays hormone concentration profiles and follicle development for one simulation of each treatment approach. Additionally, error bars at four characteristic time points (one day before treatment, one day after first drug administration, six days after first drug administration, last day of drug administration) indicate the variability in the hormone levels between 20 simulations using the same treatment conditions. The characteristic time points where chosen in a way that the results are easily comparable to the clinical data. In both cases, the FSH concentration rises with each day of the treatment. Due to the growth of multiple large follicles, which are the main source of E2, the E2 level increases significantly during ovarian stimulation. The levels are almost ten times higher compared to the normal cycle (**Figure 2C**).

Simulations of an ovarian stimulation during the luteal phase are dominated by high P4 levels during the stimulation with hMG. The high P4 concentration prevents the ovulation of follicles (through the negative feedback mechanisms of P4 on LH). The concentrations of LH, FSH, P4 and E2 in **Figures 3A–D** are comparable to observations by (26).

**Figure 3J** illustrates the follicular growth behavior under stimulation in the late follicular phase, initiated after the occurrence of a dominant follicle. The ovulation of the dominant follicle is followed by an increase in P4 concentration comparable to non-treated conditions. The E2 level decreases after the ovulation of the dominant follicle but starts to increase again. This increase is caused by multiple large follicles as a result of the stimulation.

**Figure 4:**
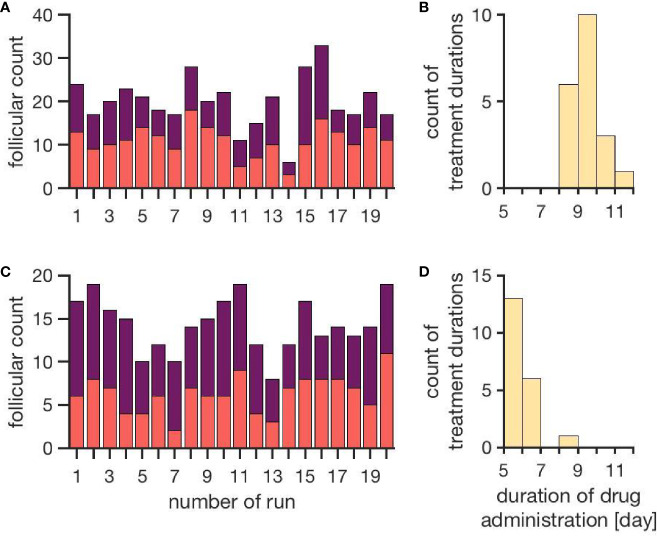
Simulation outcomes of 20 independent cycles for each treatment: ovarian stimulation induced either during the luteal phase (top, **A, B**) or the late follicular phase (bottom, **C, D**). In the upper row **(A, B)**, follicular counts and treatment duration for the luteal phase stimulation approach are displayed (red: follicles 10–14 mm; purple: follicles >14 mm). On average, 11.1 ± 3.5 follicles with a diameter of 10 to 14 mm and 8.9 ± 3.7 follicles with a diameter >14 mm are observed. The average treatment duration is 9.4 ± 0.7 days. The lower row **(C, D)** shows follicular counts and treatment durations for simulated stimulations in the late follicular phase. A treatment cycle takes about 6.0 ± 0.7 days. The average count of follicles with diameters 10 - 14 mm is 6.3 ± 2.2 and the one for follicles >14 mm is 8.0 ± 2.2. (Numbers refer to mean ± standard deviation.)

**Figure 4** represents the individual outcomes (treatment duration and follicular count) of 20 simulations per treatment protocol. The mean and standard deviation of these results are given in **Table 1**. The simulation results for ovarian stimulation initiated in the luteal phase match the observations from Kuang et al. (26). The simulated treatment duration for the late follicular phase stimulation approach is noticeably lower than the clinical observations, which goes along with comparably low counts of follicles >14 mm. **Figure 4** convincingly shows that simulations differ among each other even if non-follicular parameters are the same in all simulations. Hence, the individual growth behaviors of the follicles have a major effect on treatment simulations and outcomes.

**Table 1:** Comparison between simulation results and clinical observations.

	Luteal phase ovarian stimulation		Late follicular phase ovarian stimulation	
	Kuang et al. (26)	Simulation	Zhu and Fu (24)	Simulation
Num. of follicles with	13.9 ± 7.8	11.1 ± 3.5		6.3 ± 2.2
diameter 10 - 14 mm				
Num. of follicles with	11.1 ± 5.5	8.9 ± 3.7	11.7 ± 6.2	8.0 ± 2.2
diameter > 14 mm				
Duration of treatment	10.2 ± 1.6	9.4 ± 0.7	10.93 ± 1.66	6.0 ± 0.7
with hMG				

Ovarian stimulation is induced either during the luteal phase or the late follicular phase. Each of the two studies includes data from more than 100 woman. Patients were at the age of 18 – 40 years with a body mass index of 18 – 30 kg/m^3^. All woman showed spontaneous ovulation.

## Discussion

The mathematical model developed in this work addresses the interplay between pituitary hormones, ovarian hormones and follicular growth. Simulation results for the unstimulated cycle agree qualitatively and quantitatively with observations reported in literature. In particular:

- The time evolution of the four hormone profiles for LH, FSH, P4 and E2 is consistent with the scientific literature (56).
- An average cycle length of around 29 days, ranging from cycles with a duration of 22–25 up to 36 days, is reported in experimental studies (56–58). The simulation results are in line with these observations.
- In the literature, it is described that the variability in the length of the follicular phase is significantly higher than for the luteal phase (58, 59). The given simulation results fulfill the same property.
- The observed intra-cycle variability of 7 days is comparable to experimental results by (58).
- (32) observed the emergence of two to three waves carrying 4 to 14 follicles greater than 4 mm. The given simulation results of 16.19 ± 3.08 follicles in two waves per cycle match their experimental investigations.

The discontinuity in the profile of the E2 curve (**Figure 2** at day 85 of the simulation) is related to the growth behavior of the follicles and is caused by atresia of larger sub-dominant follicles.

By comparing the results in Table 4, it is visible that variations in the experimental data are higher than in the simulation results. That indicates the fact that the inter-individual variability in human is higher than the variability between simulations sharing one set of non-follicular parameters. The stochastic growth behavior of follicles is the only source of variability between simulations. According to (24), the LH concentration under stimulatory treatment in the late follicular phase is not supposed to increase after the ovulation of the dominant follicle due to the inhibitory effect of P4. However, this effect is not visible in the simulation results (**Figures 3F–J**). This might be due to the comparably lower P4 concentrations in the simulation results. Here, the P4 concentration at day 6 is about 0.99 ± 0.6 ng/mL, whereas the figures published by (24) indicate P4 concentrations up to more than five times as high. In the present model, the P4 concentration is linked to the formation of the corpus luteum as the only source of P4. Minor P4 sources such as the adrenal cortex are neglected. However, the equations for the P4 concentration matches experimental measurements quite well (**Figure S1** **in Supplementary Material**). A relation between the high LH concentrations, the low P4 concentrations and the follicular growth behavior are conceivable as well. Since the simulated treatment duration is several days shorter than those in the clinical observations, it appears that follicles are growing too fast during the simulation of ovarian stimulation. If this is the reason for the mismatch between the simulation results and the observations by (24), two explanations are credible: (i) the model parameters should have other values, or (ii) at least one mechanism is missing. However, at this point it was not possible to compare the simulated follicular growth under treatment to detailed experimental investigations since ultrasound measurement data were not available from literature.

Another reason for the mismatch could be that we could not simulate the clinical treatment procedures in full detail. In a clinical setting the dose is adjusted according to the treatment response, which is based on an evaluation of follicular growth during the stimulation procedure. Since the criteria for dose adjustment were not described in the available publications, we did not implement adjustments in our model.

We have not yet simulated double ovarian stimulation due to technical difficulties with the model implementation. However, we will do this in future work in order to address some of the problems that are still unsolved (60), for example the choice of the best day to start the second stimulation or the necessity of using a GnRH antagonist during the second stimulation.

Finally, we want to point out that clinical data are mainly reported as summary statistics, usually in terms of means and standard deviations, and for very few indicators, e.g. treatment duration or number and sizes of follicles on certain treatment days. However, with our model-based approach we could go beyond a simple comparison of moments. Since the model simulations generate distributions, we could compare them with data from literature if the publications about clinical trial outcomes reported the complete data distributions.

## Conclusion

This study demonstrates how mathematical modeling and simulations can contribute to enhance our mechanistic understanding of ovarian stimulation protocols. In particular, our approach allows to study the extend of variability in both treated and untreated cycles. The model simulations confirm that follicular size is not a reliable parameter for determining treatment outcome since the receptor status of each individual follicle (modeled by the FSH sensitivity threshold) and the timing of growth matter. However, we cannot (yet) make use of that knowledge in a clinical setting as long as the receptor status cannot be inferred from measurements. Making predictions on the level of individuals, either in-vivo or in-silico, will therefore remain notoriously difficult. However, models that include random effects can be used to quantify uncertainties in the predictions. Even though these uncertainties might be large, being aware of what could happen as well as identifying outliers can assist in making decisions. Moreover, the model presented here could be used to compare the outcome of different treatment strategies in terms of specific success criteria (e.g. average number of follicles larger than a threshold size at the end of the stimulation), similar to the approach in (39). This requires to first validate the model with data from other stimulation protocols. For example, in order to compare the two protocols simulated here with the three currently most often used protocols (long, short, and antagonist), we would need data on each protocol from cohorts that are comparable in terms of size and physiological stage (e.g. race, age, BMI). We therefore invite clinicians to share their data and to join interdisciplinary research projects with the ultimate goal to develop model-based clinical decision support systems.

## Data Availability Statement

The original contributions presented in the study are included in the article/**Supplementary Material**. Further inquiries can be directed to the corresponding author.

## Author Contributions

SF and SR conceived the study. ET, TM, ME, FI, TK, BL, and SR obtained the funding. BL collected the data. SF, RE, SS, BL, and SR analyzed and interpreted the data. SF, RE, SS, and SR developed the mathematical model. SF and SS implemented the model and performed the simulations. All authors contributed to the article and approved the submitted version.

## Funding

The work of SF and SR was supported by the Trond Mohn Foundation (BSF, https://www.mohnfoundation.no/), grant no. BFS2017TMT01. The funder had no role in study design, data collection and analysis, decision to publish, or preparation of the manuscript.

## Conflict of Interest

The authors declare that the research was conducted in the absence of any commercial or financial relationships that could be construed as a potential conflict of interest.

## References

[1] Mascarenhas M Flaxman S Boerma T Vanderpoel S Stevens G. National, regional, and global trends in infertility prevalence since 1990: a systematic analysis of 277 health surveys. PloS Med (2012) 9:e1001356. 10.1371/journal.pmed.1001356 23271957 PMC3525527

[2] Turner KA Rambhatla A Schon S Agarwal A Krawetz SA Dupree JM. Male infertility is a women’s health issue – Research and clinical evaluation of male infertility is needed. Cells (2020) 9:990. 10.3390/cells9040990 PMC7226946 32316195

[3] Yatsenko SA Rajkovic A. Genetics of human female infertility. Biol Reprod (2019) 101:549–66. 10.1093/biolre/ioz084 PMC8127036 31077289

[4] Wischmann T Stammer H Scherg H Gerhard I Verres R. Psychosocial characteristics of infertile couples: a study by the ‘Heidelberg Fertility Consultation Service’. Hum Reprod (2001) 16:1753–61. 10.1093/humrep/16.8.1753 11473978

[5] Quesnel-Vallée A Maximova K. Mental health consequences of unintended childlessness and unplanned births: Gender differences and life course dynamics. Soc Sci Med (2009) 68:850–7. 10.1016/j.socscimed.2008.11.012 PMC3762744 19097676

[6] Suthersan D Kennedy S Chapman M. Physical symptoms throughout ivf cycles. Hum Fertil (2011) 14:122–8. 10.3109/14647273.2011.571748 21631247

[7] Oktay K Briggs D Gosden RG. Ontogeny of follicle-stimulating hormone receptor gene expression in isolated human ovarian follicles. J Clin Endocrinol Metab (1997) 82:3748–51. 10.1210/jc.82.11.3748 9360535

[8] McGee EA Hsueh AJ. Initial and cyclic recruitment of ovarian follicles. Endocr Rev (2000) 21:200–14. 10.1210/edrv.21.2.0394 10782364

[9] Gougeon A. Regulation of ovarian follicular development in primates: facts and hypotheses. Endocr Rev (1996) 17:121–55. 10.1210/edrv-17-2-121 8706629

[10] Filicori M. The role of luteinizing hormone in folliculogenesis and ovulation induction. Fertil Steril (1999) 71:405–14. 10.1016/S0015-0282(98)00482-8 10065772

[11] Erickson GF Shimasaki S. The physiology of folliculogenesis: the role of novel growth factors. Fertil Steril (2001) 76:943–9. 10.1016/S0015-0282(01)02859-X 11704115

[12] Hiller SG Reichert JR LE Van Hall EV. Control of preovulatory follicular estrogen biosynthesis in the human ovary. J Clin Endocrinol Metab (1981) 52:847–56. 10.1210/jcem-52-5-847 6785289

[13] Henzl M Segre E. Physiology of human menstrual cycle and early pregnancy. A review of recent investigations. Contraception (1970) 1:315–38. 10.1016/0010-7824(70)90017-X

[14] Odell W. The reproductive system in women. In: DeGroot LJ, ed. Endocrinology Vol 3. New York: Grune & Stratton (1979). 3:1383–400.

[15] Franz W 3rd. Basic review: Endocrinology of the normal menstrual cycle. Prim Care (1988) 15:607. 3054966

[16] Arslan M Bocca S Mirkin S Barroso G Stadtmauer L Oehninger S. Controlled ovarian hyperstimulation protocols for in vitro fertilization: two decades of experience after the birth of Elizabeth Carr. Fertil Steril (2005) 84:555–69. 10.1016/j.fertnstert.2005.02.053 16169382

[17] Shrestha D La X Feng HL. Comparison of different stimulation protocols used in in vitro fertilization: A review. Ann Trans Med (2015) 3:137. 10.3978/j.issn.2305-5839.2015.04.09 PMC4486909 26207230

[18] Lai Q Zhang H Zhu G Li Y Jin L He L. Comparison of the GnRH agonist and antagonist protocol on the same patients in assisted reproduction during controlled ovarian stimulation cycles. Int J Clin Exp Pathol (2013) 6:1903–10. PMC3759499 24040457

[19] Huirne J Homburg R Lambalk C. Are GnRH antagonists comparable to agonists for use in IVF? Hum Reprod (2007) 22:2805–13. 10.1093/humrep/dem270 17872909

[20] Khalaf M Mittre H Levallet J Hanoux V Denoual C Herlicoviez M. GnRH agonist and GnRH antagonist protocols in ovarian stimulation: differential regulation pathway of aromatase expression in human granulosa cells. Reprod Biomed Online (2010) 21:56–65. 10.1016/j.rbmo.2010.03.017 20457540

[21] Domar A. Impact of psychological factors on dropout rates in insured infertility patients. Fertil Steril (2004) 81:271–3. 10.1016/j.fertnstert.2003.08.013 14967355

[22] Pasch LA Holley SR Bleil ME Shehab D Katz PP Adler NE. Addressing the needs of fertility treatment patients and their partners: are they informed of and do they receive mental health services? Fertil Steril (2016) 106:209–15.e2. 10.1016/j.fertnstert.2016.03.006 27018159

[23] Sighinolfi G Grisendi V La Marca A. How to personalize ovarian stimulation in clinical practice. J Turk Ger Gynecol Assoc (2017) 18:148. 10.4274/jtgga.2017.0058 28890430 PMC5590212

[24] Zhu X Fu Y. Evaluation of ovarian stimulation initiated from the late follicular phase using human menopausal gonadotropin alone in normo-ovulatory women for treatment of infertility: A retrospective cohort study. Front Endocrinol (2019) 10:448. 10.3389/fendo.2019.00448 PMC6617422 31333588

[25] Kim JH Kim SK Lee HJ Lee JR Jee BC Suh CS. Efficacy of random-start controlled ovarian stimulation in cancer patients. J Korean Med Sci (2015) 30:290–5. 10.3346/jkms.2015.30.3.290 PMC4330484 25729252

[26] Kuang Y Hong Q Chen Q Lyu Q Ai A Fu Y. Luteal-phase ovarian stimulation is feasible for producing competent oocytes in women undergoing in vitro fertilization/intracytoplasmic sperm injection treatment, with optimal pregnancy outcomes in frozen-thawed embryo transfer cycles. Fertil Steril (2014b) 101:105–11. 10.1016/j.fertnstert.2013.09.007 24161646

[27] Cakmak H Rosen M. Ovarian stimulation in cancer patients. Fertil Steril (2013) 99:1476–84. 10.1016/j.fertnstert.2013.03.029 23635348

[28] Moffat R Pirtea P Gayet V Wolf JP Chapron C de Ziegler D. Dual ovarian stimulation is a new viable option for enhancing the oocyte yield when the time for assisted reproductive technnology is limited. Reprod Biomed Online (2014) 29:659–61. 10.1016/j.rbmo.2014.08.010 25311972

[29] Kuang Y Chen Q Hong Q Lyu Q Ai A Fu Y. Double stimulations during the follicular and luteal phases of poor responders in IVF/ICSI programmes (Shanghai Protocol). Reprod Biomed Online (2014a) 29:684–91. 10.1016/j.rbmo.2014.08.009 25444501

[30] de Almeida Cardoso MC Evangelista A Sartório C Vaz G Werneck CLV Guimarães FM. Can ovarian double-stimulation in the same menstrual cycle improve IVF outcomes? JBRA Assist Reprod (2017) 21:217. 10.5935/1518-0557.20170042 28837031 PMC5574644

[31] Baerwald A Adams G Pierson R. Characterization of ovarian follicular wave dynamics in women. Biol Reprod (2003a) 69:1023–31. 10.1095/biolreprod.103.017772 12748128

[32] Baerwald A Adams G Pierson R. A new model for ovarian follicular development during the human menstrual cycle. Fertil Steril (2003b) 80:116–22. 10.1016/S0015-0282(03)00544-2 12849812

[33] Harris-Clark L Schlosser P Selgrade J. Multiple stable periodic solutions in a model for hormonal control of the menstrual cycle. Bull Math Biol (2003) 65:157–73. 10.1006/bulm.2002.0326 12597121

[34] Panza N Wright A Selgrade J. A delay differential equation model of follicle waves in women. J Biol Dyn (2016) 10:200–21. 10.1080/17513758.2015.1115564 26674178

[35] Reinecke I Deuflhard P. A complex mathematical model of the human menstrual cycle. J Theor Biol (2007) 247:303–30. 10.1016/j.jtbi.2007.03.011 17448501

[36] Reinecke I. Mathematical modeling and simulation of the female menstrual cycle. Ph.D. thesis, PhD thesis. Freie Universität Berlin (2009).

[37] Röblitz S Stötzel C Deuflhard P Jones HM Azulay D-O van der Graaf PH. A mathematical model of the human menstrual cycle for the administration of GnRH analogues. J Theor Biol (2013) 321:8–27. 10.1016/j.jtbi.2012.11.020 23206386

[38] Lange A Schwieger R Plöntzke J Schäfer S Röblitz S. Follicular competition in cows: the selection of dominant follicles as a synergistic effect. J Math Biol (2018) 78(3):579–606. 10.1007/s00285-018-1284-0 30194480

[39] Ehrig R Dierkes T Schäfer S Röblitz S Tronci E Mancini T. An Integrative Approach for Model Driven Computation of Treatments in Reproductive Medicine. ZIB report 16-04, Berlin: Zuse Institute (2016). Available at: https://opus4.kobv.de/opus4-zib/frontdoor/index/index/docId/5710.

[40] Mancini T Mari F Massini A Melatti I Salvo I Sinisi S. Computing personalised treatments through in silico clinical trials. A case study on downregulation in assisted reproduction. In: Proceedings of 25th RCRA International Workshop on Experimental Evaluation of Algorithms for Solving Problems with Combinatorial Explosion. EasyChair (2018). 10.29007/g864

[41] Tronci E Mancini T Salvo I Sinisi S Mari F Melatti I. Patient-specific models from inter-patient biological models and clinical records. In: Proceedings of 14th Conference in Formal Methods in Computer-Aided Design (FMCAD 2014). IEEE (2014). p. 207–14. 10.1109/FMCAD.2014.6987615

[42] Mancini T Tronci E Salvo I Mari F Massini A Melatti I. Computing biological model parameters by parallel statistical model checking. In: Proceedings of the 3rd International Conference on Bioinformatics and Biomedical Engineering (IWBBIO 2015) (Springer), vol. 9044 of Lecture Notes in Computer Science. Springer (2015). p. 542–54. 10.1007/978-3-319-16480-9_52

[43] Sinisi S Alimguzhin V Mancini T Tronci E Leeners B. Complete populations of virtual patients for in silico clinical trials. Bioinformatics (2020a). 10.1093/bioinformatics/btaa1026 33325489

[44] Sinisi S Alimguzhin V Mancini T Tronci E Mari F Leeners B. Optimal personalised treatment computation through in silico clinical trials on patient digital twins. Fundam Inform (2020b) 174:283–310. 10.3233/FI-2020-1943

[45] Waller K Swan SH Windham GC Fenster L Elkin EP Lasley BL. Use of urine biomarkers to evaluate menstrual function in healthy premenopausal women. Am J Epidemiol (1998) 147:1071–80. 10.1093/oxfordjournals.aje.a009401 9620051

[46] Brown J. Pituitary control of ovarian function—concepts derived from gonadotrophin therapy. Aust New Z J Obstet Gynaecol (1978) 18:47–54. 10.1111/j.1479-828X.1978.tb00011.x 278588

[47] Baerwald A Adams G Pierson R. Ovarian antral folliculogenesis during the human menstrual cycle: A review. Hum Reprod Update (2011) 18:73–91. 10.1093/humupd/dmr039 22068695

[48] Fauser BB van Heusden AM. Manipulation of human ovarian function: physiological concepts and clinical consequences. Endocr Rev (1997) 18(1):71–106. 10.1210/edrv.18.1.0290 9034787

[49] Adams G Kot K Smith C Ginther O. Selection of a dominant follicle and suppression of follicular growth in heifers. Anim Reprod Sci (1993) 30:259–71. 10.1016/0378-4320(93)90076-4

[50] Schipper I Hop WC Fauser BC. The follicle-stimulating hormone (FSH) threshold/window concept examined by different interventions with exogenous FSH during the follicular phase of the normal menstrual cycle: duration, rather than magnitude, of fsh increase affects follicle development. J Clin Endocrinol Metab (1998) 83:1292–8. 10.1210/jc.83.4.1292 9543158

[51] Baird D. The selection of the follicle of the month. In: From Ovulation to Implantation Proceedings of the VII Regnier de Graaf Symposium. Maastricht, the Netherlands: Excerpta Medica (1990).

[52] Baird DT Bäckström T McNeilly AS Smith SK Wathen CG. Effect of enucleation of the corpus luteum at different stages of the luteal phase of the human menstrual cycle on subsequent follicular development. J Reprod Fertil (1984) 70:615–24. 10.1530/jrf.0.0700615 6422035

[53] Baird D Fraser I. Concentration of oestrone and oestradiol in follicular fluid and ovarian venous blood of women. Clin Endocrinol (1975) 4:259–66. 10.1111/j.1365-2265.1975.tb01533.x 1149301

[54] McNatty K Baird D Bolton A Chambers P Corker C McLean H. Concentration of oestrogens and androgens in human ovarian venous plasma and follicular fluid throughout the menstrual cycle. J Endocrinol (1976) 71:77–85. 10.1677/joe.0.0710077 978120

[55] Dataset Kompendium. Arzneimittelkompendium der Schweiz (2020). Available at: https://compendium.ch/ (Accessed 10 June 2020).

[56] Landgren B-M Unden A-L Diczfalusy E. Hormonal profile of the cycle in 68 normally menstruating women. Eur J Endocrinol (1980) 94:89–98. 10.1530/acta.0.0940089 6770571

[57] Cole L Ladner D Byrn F. The normal variabilities of the menstrual cycle. Fertil Steril (2009) 91:522–7. 10.1016/j.fertnstert.2007.11.073 18433748

[58] Fehring RJ Schneider M Raviele K. Variability in the phases of the menstrual cycle. J Obstet Gynecol Neonatal Nurs (2006) 35:376–84. 10.1111/j.1552-6909.2006.00051.x 16700687

[59] Lenton EA Landgren B-M Sexton L. Normal variation in the length of the luteal phase of the menstrual cycle: identification of the short luteal phase. BJOG (1984) 91:685–9. 10.1111/j.1471-0528.1984.tb04831.x 6743610

[60] Sighinolfi G Sunkara SK La Marca A. New strategies of ovarian stimulation based on the concept of ovarian follicular waves: from conventional to random and double stimulation. Reprod Biomed Online (2018) 37:489–97. 10.1016/j.rbmo.2018.07.006 30170909

