# Supplementary material for: Mathematical Modeling and Simulation Provides Evidence for New Strategies of Ovarian Stimulation
Source: Front Endocrinol (Lausanne). 2021 Mar 11;12:613048. doi: 10.3389/fendo.2021.613048 (PMC8006380; doi:10.3389/fendo.2021.613048)
Supplement: Supplementary file 1 [file DataSheet_1.pdf]

Supplementary Material

1 EQUATIONS

The equations in this sections were used for the simulation of the normal menstrual cycle without drug administration. A list of parameters and short descriptions are provided in section 2 of the supplementary material. Additional explanations and biological motivations for the equations dealing with hormone dynamics (S1-19) can be found in Röblitz et al. (2013).

Hill functions are used to model stimulatory (H^+) or inhibitory (H^-) effects:

$$H^+(S, T_i^j, n_i^j) = \frac{(S/T_i^j)^{n_i^j}}{1 + (S/T_i^j)^{n_i^j}}, \qquad H^-(S, T_i^j, n_i^j) = \frac{1}{1 + (S/T_i^j)^{n_i^j}}.$$

$S(t) \geq 0$ denotes the influencing substance. The threshold $T > 0$ represents the amount of S at which the Hill function has the value $1/2$, i.e., where 50% of the maximum effect is reached. $n \geq 1$ is called the Hill coefficient and regulates the rate of switching. *i* denotes the influencing species and *j* the influenced species.

Equations S1-4 deal with LH dynamics in two compartments. LH synthesis is described by a basal synthesis rate constant b_{Syn}^{LH} and is modulated by two Hill equations (stimulation by E2 and inhibition by P4).

$$Syn_{LH}(t) = (b_{Syn}^{LH} + k_{E2}^{LH} \cdot H^+(E2(t), T_{E2}^{LH}; n_{E2}^{LH})) \cdot H^-(P4(t), T_{P4}^{LH}; n_{P4}^{LH}) \tag{S1}$$

LH is released from the pituitary with a basal release rate constant b_{Rel}^{LH}, which depends on the amount of LH in the pituitary and which is stimulated by the GnRH-receptor complex.

$$Rel_{LH}(t) = (b_{Rel}^{LH} + k_{G\text{-}R}^{LH} \cdot H^+(G\text{–}R(t), T_{G\text{-}R}^{LH}; n_{G\text{-}R}^{LH})) \cdot LH_{Pit}(t) \tag{S2}$$

The amount of LH in the pituitary can be described by the difference between the synthesized and the released amount of LH.

$$\frac{d}{dt} LH_{Pit}(t) = Syn_{LH}(t) - Rel_{LH}(t), \tag{S3}$$

The released amount of LH is diluted in the blood volume V_{blood}. Through the blood stream, LH reaches its receptor and forms complexes. LH is removed from the systems with the clearance rate constant k_{cl}^{LH}.

$$\frac{d}{dt} LH_{blood}(t) = \frac{1}{V_{blood}} \cdot Rel_{LH}(t) - k_{cl}^{LH} \cdot LH_{blood}(t). \tag{S4}$$

FSH synthesis is inhibited by progesterone and the frequency of pulsatile GnRH release.

$$Syn_{FSH}(t) = \frac{k_{p4}^{FSH}}{1 + \left(\frac{P4(t)}{T_{P4}^{FSH}}\right)^{n_{P4}^{FSH}}} \cdot H^-(freq, T_{freq}^{FSH}; n_{freq}^{FSH}) \tag{S5}$$

The fundamentals of the equations S6-8 are analogue to S2-4.

$$Rel_{FSH}(t) = (b_{Rel}^{FSH} + k_{G\text{-}R}^{FSH} \cdot H^+(G\text{-}R(t), T_{G\text{-}R}^{FSH}; n_{G\text{-}R}^{FSH})) \cdot FSH_{Pit}(t) \tag{S6}$$

$$\frac{d}{dt} FSH_{Pit}(t) = Syn_{FSH}(t) - Rel_{FSH}(t). \tag{S7}$$

$$\frac{d}{dt} FSH_{blood}(t) = \frac{1}{V_{blood}} \cdot Rel_{FSH}(t) - (k_{blood}^{FSH} - k_{cl}^{FSH}) \cdot FSH_{blood}(t). \tag{S8}$$

FSH reaches the ovaries through the blood stream and binds to its receptors on the follicular surfaces.

$$\frac{d}{dt} FSH_{foll}(t) = \frac{V_{blood}}{V_{ovaries}} \cdot k_{blood}^{FSH} \cdot FSH_{blood}(t) - (k_{on}^{FSH} \cdot R_{FSH}(t) - k_{cl}^{FSH_{foll}}) \cdot FSH_{foll}(t) \tag{S9}$$

The dynamics of the FSH receptors is described in three steps (S10-12). FSH binds to its receptor R_{FSH}, resulting in the formation of an active complex $FSH\text{-}R$. Desensitised receptors $R_{FSH,des}$ are caused by the dissociation of the complex. Each step has a characteristic reaction rate constant.

$$\frac{d}{dt} R_{FSH}(t) = k_{recy}^{FSH} \cdot R_{FSH,des}(t) - k_{on}^{FSH} \cdot FSH_{Foll}(t) \cdot R_{FSH}(t) \tag{S10}$$

$$\frac{d}{dt} FSH\text{-}R(t) = k_{on}^{FSH} \cdot FSH_{foll}(t) \cdot R_{FSH}(t) - k_{des}^{FSH} \cdot FSH\text{-}R(t) \tag{S11}$$

$$\frac{d}{dt} R_{FSH,des}(t) = k_{des}^{FSH} \cdot FSH\text{-}R(t) - k_{recy}^{FSH} \cdot R_{FSH,des}(t), \tag{S12}$$

$freq$ and $mass$ characterize the GnRH pulse generator needed to describe the GnRH dynamics in the system. The basal frequency f_0 is modulated by the stimulatory effect of E2 and the inhibitory effect of P4.

$$freq(t) = f_0 \cdot H^-(P4(t), T_{P4}^{freq}; n_{P4}^{freq}) \cdot (1 + m_{E2}^{freq} \cdot H^+(E2(t), T_{E2}^{freq}; n_{E2}^{freq})) \tag{S13}$$

The basal released mass a_0 is regulated by E2 stimulation as well as inhibition depending on the E2 concentration.

$$mass(t) = a_0 \cdot (H^+(E2(t), T_{E2}^{mass,1}; n_{E2}^{mass,1}) + H^-(E2(t), T_{E2}^{mass,2}; n_{E2}^{mass,2})) \tag{S14}$$

$$\frac{d}{dt}G(t) = mass(t) \cdot freq(t) - k_{on}^{G} \cdot G(t) \cdot R_{G,a}(t) + k_{off}^{G} \cdot G\text{-}R_a(t) - k_{degr}^{G} \cdot G(t) \tag{S15}$$

The GnRH receptor is present in four states in the model (S16-19). Free active receptors $R_{G,a}$ are available for binding GnRH with a forward rate k_{on}^{G} and a reverse rate k_{off}^{G}. The receptors and receptor complexes can switch between active and inactive states.

$$\frac{d}{dt}R_{G,a}(t) = k_{off}^{G} \cdot G(t) \cdot G\text{-}R_a(t) - k_{on}^{G} \cdot R_{G,a}(t) - k_{inter}^{R_G} \cdot R_{G,a}(t) + k_{recy}^{R_G} R_{G,i} \tag{S16}$$

$$\frac{d}{dt}R_{G,i}(t) = k_{diss}^{G\text{-}R_i} \cdot G\text{-}R_i(t) + k_{inter}^{R_G} \cdot R_{G,a}(t) - k_{recy}^{R_{G,i}} \cdot R_{G,i}(t) + k_{Syn}^{R_{G,i}} - k_{degr}^{R_{G,i}} \cdot R_{G,i}(t) \tag{S17}$$

$$\frac{d}{dt}G\text{-}R_a(t) = k_{on}^{G} \cdot G(t) \cdot R_{G,a}(t) - k_{off}^{G} \cdot G\text{-}R_a(t) - k_{inact}^{R-G} \cdot G\text{-}R_a(t) + k_{act}^{R-G} \cdot G\text{-}R_i(t) \tag{S18}$$

$$\frac{d}{dt}G\text{-}R_i(t) = k_{inact}^{G\text{-}R} \cdot R_{G,i}(t) G\text{-}R_a(t) - k_{act}^{G\text{-}R_i} \cdot -k_{degr}^{G\text{-}R_i} \cdot G\text{-}R_i(t) - k_{diss}^{G\text{-}R_i} \cdot G\text{-}R_i(t). \tag{S19}$$

The concept to simulate follicular maturation is derived from Lange et al. (2018). The Hill term in equation (S20) represents the stimulation of follicular growth and competition as soon as the individual FSH sensitivity threshold $T_{FSH\text{-}R}(i)$ is exceeded.

$$\frac{d}{dt}x_i = H^+(FSH\text{-}R, T_{FSH\text{-}R}(i), n_{FSH\text{-}R}) \cdot (\xi - x_i) x_i (\gamma - \kappa(\sum x^\nu - \mu x_i^\nu)) \tag{S20}$$

The growth rate γ is inhibited by P4 and stimulated by the FSH receptor complex,

$$\gamma = \gamma_0 \cdot H^-(P4, T_{P4}^{\gamma}, n_{P4}^{\gamma}) \cdot H^+(FSH\text{-}R, T_{FSH\text{-}R}^{\gamma}, n_{FSH\text{-}R}^{\gamma}), \tag{S21}$$

whereas κ is inhibited by the FSH receptor complex,

$$\kappa = \kappa_0 \cdot H^-(FSH, T_{FSH}^{\kappa}, n_{FSH}^{\kappa}). \tag{S22}$$

The P4 concentration in the model is described by a Gaussian with three parameters c_1, $v_{1,2}$ that characterize its height, mean and variance and whose values were determined by fitting the curve to P4

data, see Fig. S1. The mean is located with respect to the last time point of ovulation, T_{ovu}.

$$P4(t) = c_1 \cdot e^{-v_1(t-(T_{ovu}+v_2))^2}. \tag{S23}$$

We assume that all follicles with a radius larger than T_{FS} contribute to E2 production, and that the amount of E2 produced depends linearly on their surface FS.

$$FS = \pi \cdot \sum \frac{(x_i)^{n_{FS}}}{(x_i)^{n_{FS}} + (T_{FS})^{n_{FS}}} \cdot (x_i)^2. \tag{S24}$$

$$(E2)(t) = (p_0^{E2} + c_2 \cdot FS) + c_3 \cdot e^{-v_3(t-(T_{ovu}+v_4))^2}. \tag{S25}$$

The second summand represents the amount of E2 produced by the corpus luteum.

Simulating ovarian stimulation with r-FSH, which is structurally identical to FSH, is based on the idea that the administration of a drug which is identical to a species in the system can be modelled as an additional time dependent source term. The following algebraic equation is used to calculate the drug concentration $c(t)$ in the system.

$$c(t) = \frac{D \cdot \beta^2}{(\beta - c_L)^2} \cdot e^{-\beta \cdot t} \cdot (c_L \cdot t - \beta \cdot t - 2) + e^{-c_L \cdot t} \tag{S26}$$

The three parameters D, β and c_L were calculated uniquely for each administered drug from the corresponding values of three pharmacokinetic parameters given in (Kompendium, 2020). For this work, equation (S25) is used to calculate the concentration of r-FSH, which is then added to the concentration of FSH to form the total FSH concentration in the system.

2 LIST OF PARAMETERS

The following table includes all model parameters, their values and short descriptions. Hill terms and coefficients are not described in further detail, since they are represented in a consistent notation which is introduced at the beginning of the first section (1 EQUATIONS) of the supplementary material. Parameters marked with * are either newly introduced or modified compared to Röblitz et al. (2013).

Symbol	Value	Unit	Explanation
b_{Syn}^{LH} *	1827.48	IU/d	LH synthesis rate constant
k_{E2}^{LH}	7309.92	IU/d	reaction rate constant referring to the stimulatory effect of E2 on the LH synthesis
T_{E2}^{LH}	192.2	pg/mL	
n_{E2}^{LH}	10	–	
T_{P4}^{LH}	2.371	ng/mL	
n_{P4}^{LH}	1	–	
b_{Rel}^{LH}	0.00476	1/d	release rate constant of LH into the blood
$k_{G\text{-}R}^{LH}$	0.1904	1/d	reaction rate constant of the stimulatory effect of GnRH receptor complex on the LH release
$T_{G\text{-}R}^{LH}$ *	0.001	nmol/L	
$n_{G\text{-}R}^{LH}$	5	–	
V_{blood} *	5.5	L	blood volume
k_{cl}^{LH}	74.851	1/d	clearance rate constant of LH
T_{freq}^{FSH}	12.8	1/d	
n_{freq}^{FSH}	5	–	
T_{P4}^{FSH} *	5	ng/mL	
n_{P4}^{FSH} *	2	–	
b_{Rel}^{FSH}	0.057	1/d	FSH release rate constant
$k_{G\text{-}R}^{FSH}$	0.272	1/d	reaction rate constant of the stimulatory effect of the GnRH-receptor complex on the FSH release
$T_{G\text{-}R}^{FSH}$	0.0003	nmol/L	
$n_{G\text{-}R}^{FSH}$	2	–	
k_{blood}^{FSH} *	0.02	1/d	diffusion rate constant of FSH from the blood to the ovaries
k_{on}^{FSH}	3.529	L/(d·IU)	FSH receptor bindung rate constant
k_{cl}^{FSH}	114.25	1/d	FSH clearance rate constant
k_{recy}^{FSH}	61.029	1/d	FSH receptor recycling rate constant
k_{des}^{FSH}	138.3	1/d	FSH receptor desensitisation rate constant
$k_{cl}^{FSH_{Foll}}$ *	0.1	1/d	FSH clearance rate constant at the follicles

Symbol	Value	Unit	Explanation
$V_{ovaries}$ *	0.011	L	ovarian volume
f_0	16	1/d	basal GnRH pulse frequency
T_{P4}^{freq}	1.2	ng/mL	
n_{P4}^{freq}	2	–	
m_{E2}^{freq}	1	–	GnRH pulse amplitude
T_{E2}^{freq}	220	pg/mL	
n_{E2}^{freq}	10	–	
a_0	5.593e-3	nmol	basal GnRH pulse amplitude
$T_{E2}^{mass,1}$	220	pg/mL	
$n_{E2}^{mass,1}$	2	–	
$T_{E2}^{mass,2}$	9.6	pg/mL	
$n_{E2}^{mass,2}$	1	–	
k_{degr}^{G}	0.447	1/d	GnRH receptor degradation rate constant
k_{on}^{G}	322.18	$\frac{L}{d \cdot nmol}$	GnRH receptor binding rate constant
k_{off}^{G}	644.35	1/d	GnRH-receptor complex reverse-rate constant
$k_{degr}^{G\text{-}R_i}$	0.00895	1/d	inactive GnRH-receptor complex degradation rate constant
$k_{diss}^{G\text{-}R_i}$	32.218	1/d	inactive GnRH-receptor complex dissociation rate constant
$k_{inter}^{R_G}$	3.222	1/d	GnRH receptor inactivation rate constant
$k_{recy}^{R_G}$	32.218	1/d	GnRH receptor recycling rate constant
$k_{degr}^{R\text{-}G}$	0.0895	1/d	GnRH-receptor complex degradation rate constant
$k_{inact}^{G\text{-}R}$	32.218	1/d	GnRH-receptor complex inactivation rate constant
$k_{degr}^{R_{G,i}}$	3.222	1/d	inactive GnRH receptor degradation rate constant
$k_{syn}^{R_{G,i}}$	8.949e-5	$\frac{nmol}{L \cdot d}$	GnRH receptor synthesis rate constant
ξ *	25	mm	maximal follicle size
κ_0 *	0.000048	–	competition strength
$T_{FSH\text{-}R}^{\kappa}$ *	0.85	nmol/mL	
$n_{FSH\text{-}R}^{\kappa}$ *	25	–	
ν *	2	–	fractal dimension
μ *	1	–	self-harm
γ_0 *	0.03	mm/d	basal growth rate
T_{P4}^{γ} *	0.33	ng/mL	
n_{P4}^{γ} *	5	–	
$T_{FSH\text{-}R}^{\gamma}$ *	0.45	nmol/L	
$n_{FSH\text{-}R}^{\gamma}$ *	10	–	

Symbol	Value	Unit	Explanation
$T_{FSH\text{-}R}$ *	0.85	nmol/L	
$n_{FSH\text{-}R}$ *	25	–	
c_1 *	13	–	scaling factor for P4 production
c_2 *	0.02	pg/mL	scaling factor for follicular surface effect in E2 production
c_3 *	150	–	scaling factor for E2 production
v_1 *	0.06	–	
v_2 *	7	d	
v_3 *	1	–	
v_4 *	0.02	d	
T_{FS} *	15	mm	follicular size threshold for E2 production
n_{FS} *	5	–	
p_0^{E2} *	1	pg/mL	
D^{FSH} *	5.0201	IU/d	Menopur administartion 225 IU
β^{FSH} *	9.87	1/d	Menopur administartion 225 IU
c_L^{FSH} *	0.42	1/d	Menopur administartion 225 IU
D^{LH} *	2.14	IU/d	Menopur administartion 225 IU
β^{LH} *	6.04	1/d	Menopur administartion 225 IU
c_L^{LH} *	3.199	1/d	Menopur administartion 225 IU

3 PROGESTERONE CURVE

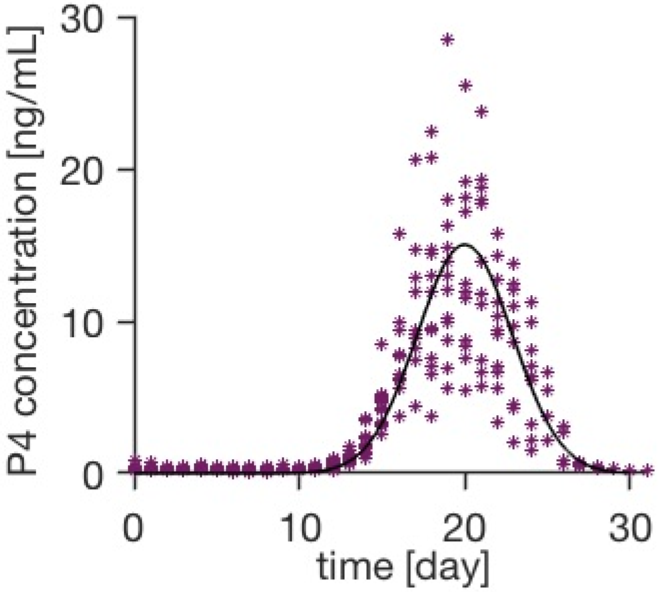

Figure S1. Fit of Gaussian curve to P4 serum concentration measurement in woman provided by Pfizer ltd. The measurement points are represented in purple. The black line presents the progesterone curve as it is implemented in the model.

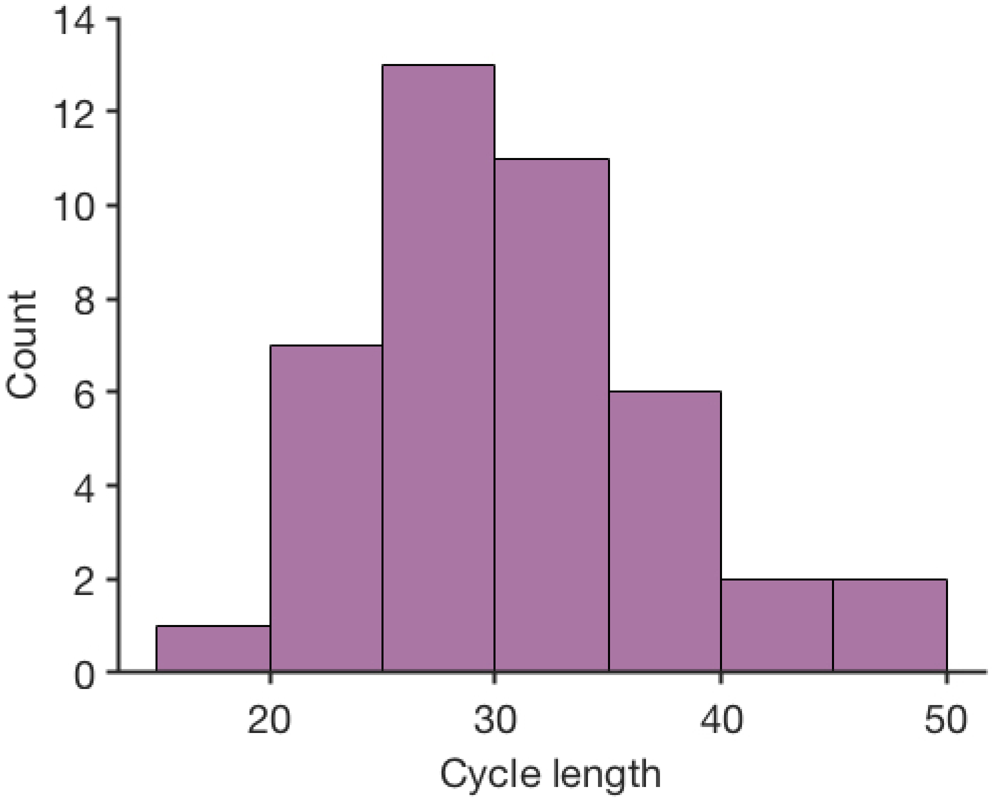

Figure S2. Representation of the cycle length of 42 simulated menstrual cycles.
